# 86185 Food Cost and Perceptions: through the lens of coaches providing family-based childhood obesity treatment

**DOI:** 10.1017/cts.2021.552

**Published:** 2021-03-30

**Authors:** Melissa Ramel, Denise Wilfley, Rachel Tabak

**Affiliations:** Washington University in St. Louis School of Medicine

## Abstract

ABSTRACT IMPACT: This work will help to identify ways to adapt family-based obesity treatment based on families’ food purchasing behaviors and beliefs. OBJECTIVES/GOALS: Families in obesity treatment are encouraged to make dietary changes. Dietary changes are impacted by food choices, which can be influenced by food cost. The objective of this research is to explore families’ food purchase behaviors and beliefs from the perspective of their health coach, and to assess how health coaches adapt treatment to address these. METHODS/STUDY POPULATION: Semi- structured telephone interviews were conducted with 10 health coaches in the Effectiveness of Family-Based Weight Loss Treatment Implementation in Primary Care (PLAN) study across four geographic locations in MO, NY, and OH. Topics covered were professional background, perspectives on working with families, and discussions with families regarding cost perception and food choice. Conventional content analysis was used through ‘open-coding’ of transcribed text by reading the transcripts and assigning labels. Codes were then organized into themes. In addition to the interviews, coaches were asked to complete a FRAME checklist to identify adaptations or modifications that were made to the treatment. RESULTS/ANTICIPATED RESULTS: The coaches reported that cost is a barrier to making healthier food choices for some but not all of their FBT families. Themes for cost as a barrier include: fast food is cheaper; justification to choose old food choices; sales on foods high in calories and sugar; bulk buying; and fewer sales on healthier options. Themes for what families consider when purchasing healthier items include: perishable foods, increased waste, picky kids, lack of knowledge about healthy eating on a budget, afraid of including new foods, and no money for new foods. The final stage of content analysis for the FRAME schematic checklist is ongoing. DISCUSSION/SIGNIFICANCE OF FINDINGS: The results exemplify that families’ have different food purchasing behaviors and beliefs and consider a variety of factors when making food choices. The data gathered from the FRAME checklists will help in characterizing the adaptations or modifications made by coaches and allow for better understanding of the impact on the families.

